# The Nuclear Inclusion a (NIa) Protease of Turnip Mosaic Virus (TuMV) Cleaves Amyloid-β

**DOI:** 10.1371/journal.pone.0015645

**Published:** 2010-12-20

**Authors:** Hye-Eun Han, Saravanan Sellamuthu, Bae Hyun Shin, Yong Jae Lee, Sungmin Song, Ji-Seon Seo, In-Sun Baek, Jeomil Bae, Hannah Kim, Yung Joon Yoo, Yong-Keun Jung, Woo Keun Song, Pyung-Lim Han, Woo Jin Park

**Affiliations:** 1 Department of Life Science, Gwangju Institute of Science and Technology (GIST), Gwangju, Republic of Korea; 2 School of Biological Sciences, Seoul National University, Seoul, Republic of Korea; 3 Division of Nano Sciences and Brain Disease Research Institute, Ewha Womans University, Seoul, Republic of Korea; Universidade Federal do Rio de Janeiro, Brazil

## Abstract

**Background:**

The nuclear inclusion a (NIa) protease of turnip mosaic virus (TuMV) is responsible for the processing of the viral polyprotein into functional proteins. NIa was previously shown to possess a relatively strict substrate specificity with a preference for Val-Xaa-His-Gln↓, with the scissile bond located after Gln. The presence of the same consensus sequence, Val^12^-His-His-Gln^15^, near the presumptive α-secretase cleavage site of the amyloid-β (Aβ) peptide led us to hypothesize that NIa could possess activity against Aβ.

**Methodology/Principal Findings:**

Western blotting results showed that oligomeric as well as monomeric forms of Aβ can be degraded by NIa *in vitro*. The specific cleavage of Aβ was further confirmed by mass spectrometry analysis. NIa was shown to exist predominantly in the cytoplasm as observed by immunofluorescence microscopy. The overexpression of NIa in B103 neuroblastoma cells resulted in a significant reduction in cell death caused by both intracellularly generated and exogenously added Aβ. Moreover, lentiviral-mediated expression of NIa in APP_sw_/PS1 transgenic mice significantly reduced the levels of Aβ and plaques in the brain.

**Conclusions/Significance:**

These results indicate that the degradation of Aβ in the cytoplasm could be a novel strategy to control the levels of Aβ, plaque formation, and the associated cell death.

## Introduction

Alzheimer's disease (AD) is a progressive neurodegenerative disorders which affects approximately twenty four million people worldwide, and it is the most common form of dementia among older people. AD is characterized by progressive memory impairment and cognitive dysfunction. A distinct hallmark of AD is the deposition of amyloid plaques which are mainly composed of amyloid β (Aβ) of 40, 42, and 43 amino acids in length. Aβ is produced by the sequential cleavage of the amyloid β precursor protein (APP) by β- and γ-secretases[1], [2].

Aβ can exist in different forms such as monomers, oligomers (dimer, trimer, and tetramer), proto-fibrils, and fibrils, and these different conformational states are related to its toxicity. Oligomeric Aβ was shown to be approximately 10- and 40-fold more cytotoxic than fibrillar and monomeric Aβ, respectively[3]. A recent report also found that dimeric Aβ are 3-fold more toxic than monomeric Aβ, and that trimeric and tetrameric Aβ are up to 13-fold more toxic[4].

Although Aβ unquestionably plays a causative role in AD, the underlying mechanisms by which it contributes to the development of this disease are still controversial. It is widely accepted that Aβ exerts its pathological activity extracellularly. In pathological AD brains, Aβ is secreted into the extracellular space forming amyloid plaques[5]. When added into the culture media, Aβ can induce cell death *in vitro* in a variety of cell types[3], [4], [6]. However, accumulating evidence suggests that intracellular Aβ activity is also critical for the development of AD. Several authors have reported the intracellular localization of Aβ in the brain tissues of post-mortem AD patients and in transgenic AD mice[1], [7], [8]. A closer examination with electron microscopy and immunocytochemistry revealed that Aβ is present in diverse subcellular organelles in neuronally differentiated P19 cells, including early endosomes, trans-Golgi network, rough endoplasmic reticulum, outer mitochondrial membrane, and nuclear envelope[9]. In a triple transgenic AD mouse model, early cognitive impairments correlated with the accumulation of intracellular Aβ in the hippocampus and amygdala, without the apparent deposition of amyloid plaques or neurifibrillary tangles[10]. Intracellular Aβ was also shown to induce p53-dependent neuronal cell death[11], [12] through the impairment of mitochondrial function[13]. The intra-hippocampal injection of an antibody directed against Aβ reduced not only extracellular Aβ deposits, but also intracellular Aβ accumulation. Upon dissipation of this antibody, the re-appearance of the extracellular deposits was preceded by the accumulation of intracellular Aβ. These observations suggest that a dynamic exchange between intracellular and extracellular Aβ exists, and that intracellular Aβ serves as a source of extracellular amyloid deposits, implying a role for intracellular Aβ in the pathogenesis of AD[14], [15].

There are currently no methods proven to efficiently remove accumulated amyloids with improved AD symptoms. Since the accumulation of Aβ is considered to be the most critical single event in the pathogenesis of AD, a catabolic elimination of Aβ from the brain would be a valuable therapeutic strategy. Several proteases, including neprilysin (NEP), insulin degrading enzyme, endothelin-converting enzyme, and uPA/tPA-plasmin, have been identified for their ability to degrade Aβ[16], with NEP being the best-characterized one. The pharmacological inhibition or genetic ablation of NEP in mice has been shown to result in an increased Aβ deposition, accompanied by deficits in synaptic plasticity and an impairment in hippocampus-dependent memory[17], [18], while the viral or transgene-mediated overexpression of NEP reduced Aβ deposition and its associated cytopathology[19], [20]. However, it was recently shown that NEP overexpression did not reduce the oligomeric Aβ levels or improve deficits in learning and memory. These results appear to suggest that the NEP-dependent degradation of Aβ affected plaques more efficiently than oligomeric Aβ[21].

We have previously reported that the nuclear inclusion a (NIa) protease of Turnip mosaic virus (TuMV) contains a strict substrate specificity with a preference for Val-Xaa-His-Gln↓, with the scissile bond located after Gln[22]. Based on the fact that Aβ contains an amino acid sequence, Val-His-His-Gln, in the vicinity of the presumed α-secretase cleavage site, we tested whether NIa can cleave Aβ. In this study, we show that NIa indeed cleaves monomeric and oligomeric Aβ and that it significantly ameliorates the Aβ-induced cell death in neuronal culture cells and the Aβ-related pathology in transgenic AD mice. NIa might therefore provide a novel strategy for the clearance of toxic oligomeric Aβ from the brain of AD patients.

## Results

### Cleavage of monomeric and oligomeric Aβ by NIa

**Figure 1:**
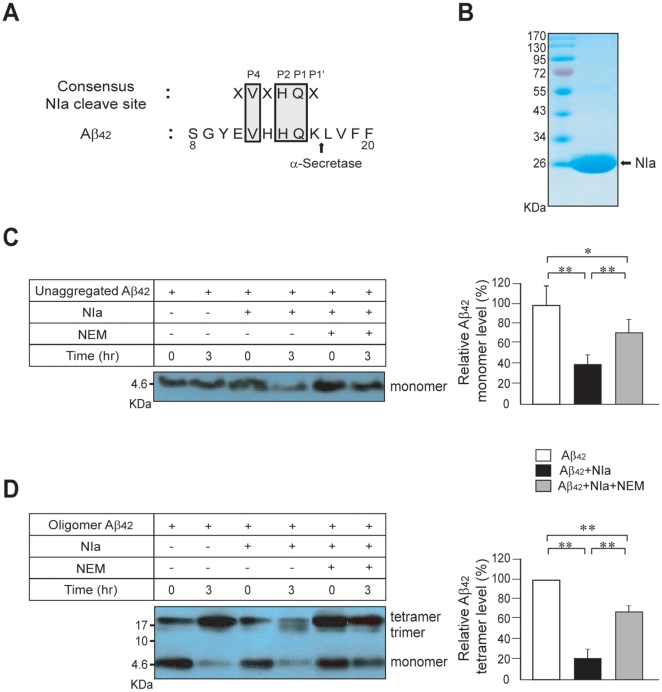
Cleavage of Aβ by NIa. (A) The amino acid sequence of Aβ is aligned with the consensus cleavage site of NIa, Val-Xaa-His-Gln. (B) NIa was purified from *E. coli* and separated by SDS-PAGE. Lane 1, molecular size markers; lane 2, NIa (10 µg). (C) Monomeric Aβ (2.5 µM) was incubated with NIa (1.5 µM) in the presence or absence of NEM(cysteine protease inhibitor) for 3 hrs at 25°C. The reaction mixture was separated on a Tris-tricine gel, blotted, and probed with the anti-Aβ antibody, 6E10. The density of each Aβ band was quantified by densitometry. The band intensities after 3 hr incubation (lanes 2, 4, and 6) were plotted relative to the band intensities of each sample at 0 hr (lanes 1, 3, and 5). n = 4. (D) Oligomeric Aβ (2.5 µM) was incubated with NIa (1.5 µM) in the presence or absence of NEM for 3 hrs at 25°C. The reaction mixture was separated and immunoblotted with anti-Aβ antibody, 6E10. The density of oligomeric Aβ bands was quantified by densitometry. The band intensities of oligomeric Aβ after 3 hr incubation (lanes 2, 4, and 6) were plotted relative to the band intensity of the Aβ only sample at the 3 hr incubation time point (lane 2). n = 4. Error bars represent SD. *p<0.05 and **p<0.01.

We have previously reported that NIa possesses a highly strict substrate specificity, with its cleavage sites defined by the conserved sequence motif Val-Xaa-His-Gln↓, in which the scissile bond is located after Gln. Interestingly, the sequence Val-His-His-Gln is present in Aβ in the vicinity of the presumed α-secretase cleavage site (Fig. 1A). Based on this finding, we aimed to determine whether NIa can specifically cleave Aβ. For this purpose, a recombinant NIa protein was expressed in *E. coli* and purified to near homogeneity on a chitin bead column (Fig. 1B). NIa was then incubated with a monomeric Aβ preparation for 3 hrs in the presence or absence of the cysteine protease inhibitor, NEM. Analysis by Western blotting revealed that the monomeric Aβ level was greatly reduced by NIa (Fig. 1C, lane 2 vs. 4), which was partially reversed in the presence of NEM (Fig. 1C, lane 6). The results of the densitometry analysis showed that NIa reduced Aβ levels by 64% in the absence of NEM and 33% in the presence of NEM, suggesting the specific cleavage of monomeric Aβ by NIa. Our findings show that NEM did not completely inhibit NIa activity, which is consistent with a previous report showing that mutations of cysteine residues in the catalytic triad of NIa did not completely abolish its proteolytic activity[23].

We then tested whether NIa is capable of cleaving oligomeric Aβ, which is known to be more toxic than monomeric Aβ. Oligomeric Aβ was prepared by incubating a solution of Aβ peptides at 4°C for 36 hrs. As assessed by SDS-PAGE, the oligomeric Aβ preparation contained roughly equal amounts of monomeric and oligomeric Aβ (Fig. 1D, lanes 1, 3, and 5), a balance that shifted toward an increase in the formation of oligomeric Aβ at the expense of monomeric Aβ after an additional 3 hour incubation at 25°C. This is consistent with a previous report showing that Aβ oligomerization was accelerated by an increase in incubation time and temperature[24]. Under the same conditions, the amount of oligomeric Aβ was greatly reduced by NIa (lane 4) as quantified by densitomeric assessment, which showed that only 19% of oligomeric Aβ remained. This NIa-mediated reduction of oligomeric Aβ was significantly blocked by NEM (lane 6) implying that NIa specifically cleaves Aβ.

**Figure 2:**
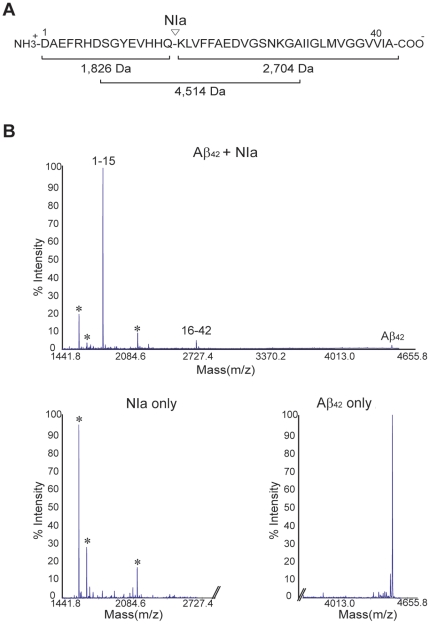
Mass spectra of monomeric Aβ incubated with NIa. (A) The calculated molecular masses of the expected cleavage products are shown. (B) Monomeric Aβ (2.5 µM) was incubated with NIa (1.5 µM) for 3 hrs at 25°C and analyzed using MALDI-TOF/TOF mass spectrometry. Note that two peaks corresponding to the Aβ cleavage products as well as a peak corresponding to Aβ were detected. As controls, NIa and Aβ were analyzed separately. Three minor peaks marked by asterisks represent contamination of the NIa preparation.

To further analyze the specific cleavage of Aβ by NIa, the cleavage products were analyzed by MALDI-TOF/TOF mass spectrometry (Fig. 2). The monomeric Aβ preparation produced a single peak without contamination, whereas NIa produced three contaminating peaks. In the reaction mixture including Aβ and NIa, the peak corresponding to Aβ was greatly reduced and two new peaks were detected (Fig. 2B), with molecular weights of 1,826 Da and 2,704 Da, corresponding to amino acids1–15 and 16–42 of Aβ, respectively (Fig. 2A). This result indicates that NIa cleaves the peptide bond after Gln^15^, as expected.

### Subcellular localization of NIa

**Figure 3:**
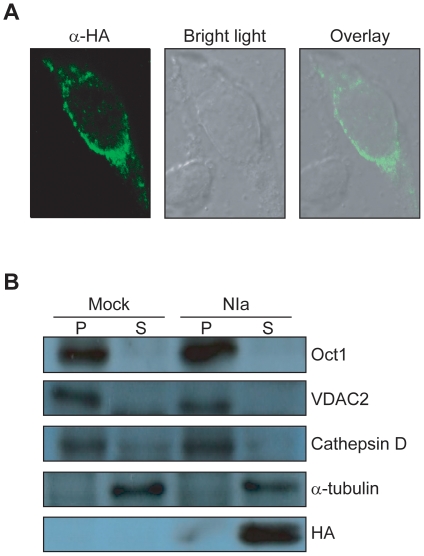
Subcellular localization of NIa in B103 neuroblastoma cells. (A) B103 neuroblastoma cells transformed with pcDNA-HA-NIa were immunostained with anti-HA antibody and observed under a confocal microscope. (B) B103 cells transformed with a blank plasmid (Mock) or pcDNA-HA-NIa (NIa) were fractionated into particulate (P) and soluble (S) fractions by differential centrifugation. The two fractions were separated by SDS-PAGE, blotted, and probed with antibodies against Oct1 (nuclear), VDAC2 (mitochondrial), cathepsin D (lysosomal), α-tubulin (cytosolic), and HA (NIa).

B103 neuroblastoma cells were transformed with an HA-tagged NIa expression vector and stained with an anti-HA antibody. Examination with confocal microscopy revealed that NIa was expressed predominantly in the cytoplasm (Fig. 3A). The transformed cells were fractionated into the particulate (P) and soluble (S) fractions and subjected to Western blotting (Fig. 3B). While Oct1 (nuclear marker), VDAC2 (mitochondrial marker), and cathepsin D (lysosomal marker) were found in the particulate fraction, HA was colocalized with α-tubulin (cytosolic marker) exclusively to the soluble fraction. These data suggest that NIa resides predominantly in the cytosol.

### NIa prevents Aβ-induced cell death

**Figure 4:**
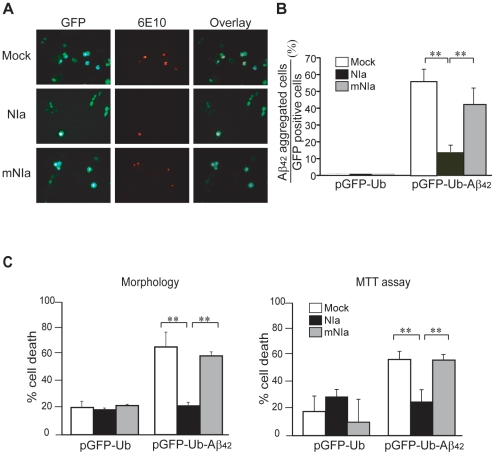
Degradation of intracellular Aβ and inhibition of intracellular Aβ-induced cell death by NIa. (A) B103 neuroblastoma cells were cotransfected with pGFPUb-Aβ and an empty vector (Mock), pcDNA-HA-NIa (NIa), or pcDNA-HA-mNIa (mNIa). After 48 hrs of incubation, the cells were immunostained with the anti-Aβ antibody, 6E10. (B) The number of Aβ-positive cells (red) and GFP-expressing cells (green) were counted under the microscope and their ratio was calculated. n = 6. (C) Cell death induced by intracellular Aβ peptide was measured by morphological and MTT assays. n = 6. Error bars represent SD. **p<0.01.

To test whether NIa possesses activity against Aβ within cells, we generated Aβ intracellularly using the plasmid pGFPUb-Aβ, encoding a triple fusion protein of green fluorescent protein (GFP), ubiquitin (Ub), and Aβ. The peptide bond between Ub and Aβ is cleaved quickly by endogenous deubiquitinating enzymes, generating an equimolar ratio of GFP-Ub and Aβ in the cytosol[25]. B103 cells were co-transformed with pGFPUb-Aβ and an empty plasmid, a NIa-expression plasmid, pcDNA-HA-NIa, or a mutant NIa expression plasmid, pcDNA-HA-mNIa. The NIa mutation consisted of an Asp to Ala substitution in the catalytic triad. The cells were then immunostained with the anti-Aβ antibody, 6E10 (Fig. 4A and B). The results revealed that the proportion of Aβ-positive cells was 56% of the total of GFP-positive cells in those cells harboring pGFPUb-Aβ and an empty plasmid (Mock), whereas the ratio sharply declined to 14% in cells harboring pGFPUb-Aβ and pcDNA-HA-NIa (NIa). The observed ratio in those cells expressing a mutant NIa protease plasmid (mNIa) was 42%, which was not significantly different from that obtained with an empty plasmid. These data indicate that NIa can degrade intracellular overexpressed Aβ.

**Figure 5:**
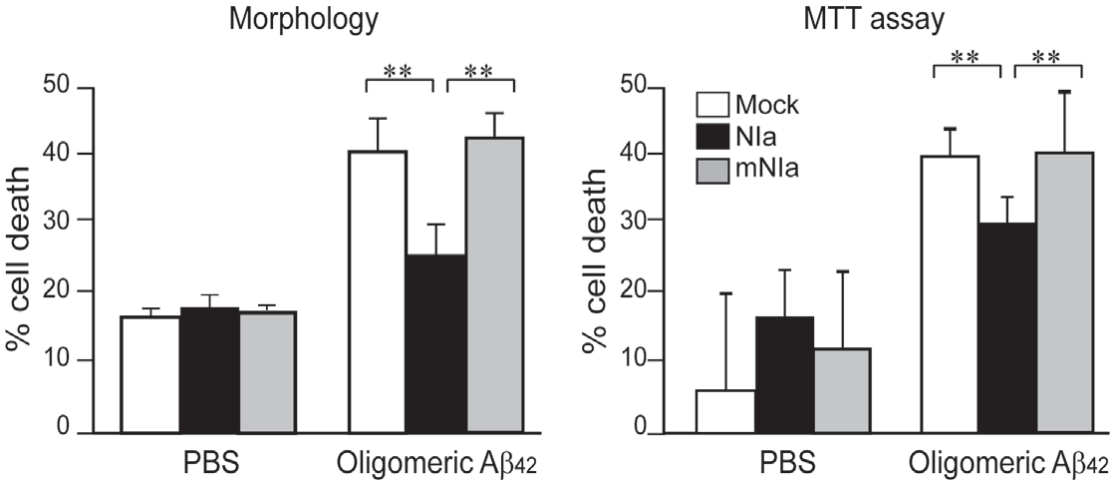
Inhibition of exogenously added Aβ-induced cell death by NIa. B103 neuroblastoma cells transfected with an empty vector (Mock), pcDNA-HA-NIa (NIa), or pcDNA-HA-mNIA (mNIa) were treated with Aβ (5 µM) in culture media for 48 hrs. Cell death was measured by morphological and MTT assays. n = 6. Error bars represent SD. **p<0.01.

To evaluate whether NIa prevents Aβ-induced cell death, we used two different methods, a morphological approach and the MTT cell viability assay (Fig. 4B and C). Intracellular expression of Aβ via pGFPUb-Aβ resulted in a significant increase in cell death (62% by the morphological assay and 55% by the MTT assay). This intracellular Aβ-induced cell death was almost completely blocked by co-transfomation with pcDNA-HA-NIa but it was not affected in cells co-expressing pcDNA-HA-mNIa. Treatment of B103 cells with exogenous Aβ also resulted in a considerable proportion of cell death (40% by the morphological assay and 38% by the MTT assay), which was inhibited by co-transfomation with pcDNA-HA-NIa but not by pcDNA-HA-mNIa co-expression (Fig. 5A and B). It was previously shown that extracellular Aβ is internalized by cell surface receptors and detected in subcellular organelles such as lysosomes, mitochondria and cytosol, causing cell death through dysfunction of these organelles[26]–[29]. It appears that cytosolic NIa can cleave internalized Aβ, although it is unknown whether NIa and internalized Aβ are co-localized. Nonetheless, our data indicate that NIa can prevent cell death induced by both intracellularly expressed and exogenously added Aβ.

### Lentiviral-mediated overexpression of NIa

**Figure 6:**
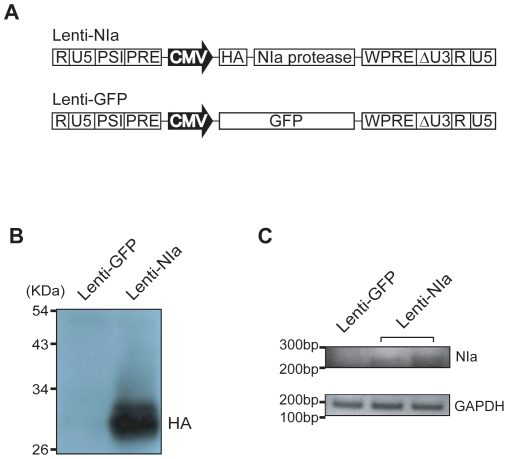
Lentiviral-mediated expression of NIa. (A) Lentiviral constructs for the expression of HA-NIa and GFP. (B) Western blotting with anti-HA antibody showed the NIa expression levels in 293T cells infected with Lenti-NIa. (C) The brains infused with Lenti-GFP and Lenti-NIa were subjected to RT-PCR. A PCR product corresponding to NIa was detected. GAPDH was used as control.

Lentiviral vectors expressing NIa and GFP were generated (Fig. 6A). Human 293T cells infected with Lenti-NIa showed a strong NIa expression, as assessed by Western blotting with anti-HA antibody (Fig. 6B). Double transgenic mice (APPswe/PS1dE9) were stereotaxically injected with 3 µl of Lenti-NIa (1×10^8^ TU) into the lateral ventricles. To evaluate the expression of NIa, immunohistochemistry was performed one month after injection. The NIa expression was detected in sections of mice injected with Lenti-NIa compared with the brain sections of control non-injected mice. The pattern of NIa expression showed a wide distribution throughout the brain including the cerebral cortex, hippocampus, amygdala, and thalamus (data not shown). RT-PCR also showed the presence of the NIa transcripts in the Lenti-NIa-infected brain. The GAPDH signal served as a control and was equally expressed in all samples (Fig. 6C).

### Decreased Aβ levels in the brain of APP_sw_/PS1 transgenic mice infused with Lenti-NIa

**Figure 7:**
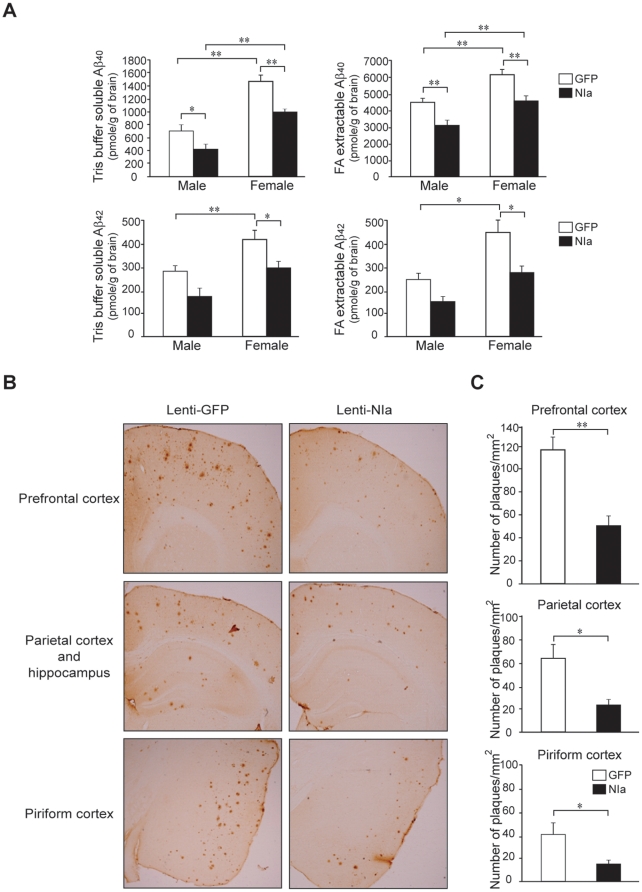
NIa-mediated reduction in Aβ levels and Aβ plaques in APP_sw_/PS1dE9 mouse brains. (A) Brains of APP_sw_/PS1dE9 were bilaterally infused with Lenti-GFP and Lenti-NIa, and the amounts of Aβ_1−40_ and Aβ_1−42_ were measured by ELISA. The amounts of soluble and insoluble Aβ_1−40_ are shown (upper lane). The amounts of soluble and insoluble Aβ_1−42_ are shown (lower lane). (B) Sections of prefrontal cortex, parietal cortex, hippocampus, and piriform cortex of APP_sw_/PS1dE9 male mouse infused with Lenti-GFP and Lenti-NIa were stained with anti-Aβ antibody (Bam-10). (C) The number of plaques in the prefrontal cortex, parietal cortex, and piriform cortex of APP_sw_/PS1dE9 male mouse infused with Lenti-GFP and Lenti-NIa was counted. For Lenti-GFP infusions, n = 5 for male and n = 5 for female. For Lenti-NIa infusions, n = 6 for male and n = 3 for female. Error bars represent SD. *p<0.05 and **p<0.01.

To assess if NIa causes a reduction in the Aβ levels in mouse brains, Lenti-NIa was infused into the lateral ventricles of the brain of APP_sw_/PS1dE9 mice at 6.5 months of age. As a control, equal amounts of Lenti-GFP were infused in the same manner. The brains were removed one month after injection and the Aβ levels in both soluble (Tris-buffer extractable) and insoluble (FA-buffer extractable) fractions were measured by ELISA. We found that the levels of both Aβ_1−40_ and Aβ_1−42_ were significantly reduced in both the soluble and insoluble factions of Lenti-NIa-infused brain when compared to the Lenti-GFP-infused brain (Fig. 7A). The Lenti-NIa infusion reduced the soluble Aβ_1−40_ by 33% in males and by 36% in females, and the insoluble Aβ_1−40_ by 24% in males and by 21% in females (Fig. 7A, upper lane). NIa also reduced the soluble Aβ_1−42_ by 38% in males and by 28% in females, and the insoluble Aβ_1−42_ by 33% in males and by 36% in females (Fig. 7A, lower lane). The reduction of Aβ_1−42_ levels in the male brains was not statistically significant.

### Reduced Aβ deposition in the brain of APP_sw_/PS1 transgenic mice infused with Lenti-NIa

Immunohistochemical analysis revealed that the Aβ deposition in the prefrontal cortex, parietal cortex, hippocampus and piriform cortex was remarkably decreased in the brain infused with Lenti-NIa in comparison to the brain infused with Lenti-GFP (Fig. 7B). Quantitative assessment of Aβ levels indicated that the Lenti-NIa infusion reduced the plaques by 58% in the prefrontal cortex, by 62% in the parietal cortex, and by 59% in the piriform cortex (Fig. 7C).

## Discussion

The generation and accumulation of Aβ is the most critical event in the development of AD, suggesting that the clearance of Aβ could provide a valuable strategy for the treatment of AD. Although Aβ exits in several assembly and aggregation forms, oligomeric Aβ is known to be the most toxic form. Aβ is oligomerized intracellularly soon after it is generated, and these molecules are then secreted from the cell. Some of the secreted Aβ oligomers enter the cell through selective uptake and subsequently cause the dysfunction of subcellular organelles, which is associated with the memory and cognitive decline typically observed in AD patients[30].

Aβ is detected in both intraneuronal cells and in the extracellular space of AD brains. Recent studies have demonstrated that intracellular Aβ levels decrease as extracellular plaques start to build up in patients with AD and in AD transgenic mouse models[10], [31]. These results suggest that the accumulation of intracellular Aβ precedes the formation of extracellular Aβ deposits in the progression of the disease. Interestingly, in cells expressing the AD-associated mutant APP, Aβ is kept within the cells, whereas in cells expressing wild-type APP, Aβ is mostly found to be secreted[32]. In addition, in aged mice carrying mutant presenilin 1, Aβ aggregation is detected within neurons, but it is absent in the extracellular fluid[33]. The inhibition of proteasome activity leads to higher levels of Aβ both *in vivo* and *in vitro*, suggesting that the proteasome is responsible for the processing of Aβ in the cytosol[25], [34], [35]. The overproduction of Aβ results in an overload of the proteasome, ultimately leading to an impairment of proteasome activity, a characteristic of AD[36], [37]. These reports support a central role for intracellular Aβ in the pathogenesis of AD.

The enhanced proteosomal activity caused by the plant polyphenol resveratrol was shown to reduce intracellular as well as extracellular Aβ levels and to prevent neurodegenerative disorders[38]. Parkin is an E3 ligase which participates in the ubiquitination of intracellularly expressed Aβ. The overexpression of parkin was found to result in a proteasome-mediated reduction of Aβ levels[39], whereas the knockout of parkin caused an accumulation of Aβ deposits[39], [40]. Enhanced clearance of intracellular Aβ may therefore prevent plaque formation, secondary pathology and premature death.

In this study, we show that a plant viral protease, NIa, specifically cleaves oligomeric as well as monomeric Aβ *in vitro* and is predominantly localized in the cytosol of neuronal cells. The expression of NIa in neuronal cells inhibits cell death induced both by intracellularly expressed and exogenously added Aβ. In addition, lentiviral-mediated overexpression of NIa in the brain of AD transgenic mice was found to reduce the levels of Aβ and plaque formation. These data provide additional evidence supporting a critical role for intracellular Aβ in the pathogenesis of AD. In this regard, NIa could be used as a novel tool to study the molecular events underlying the induction of cell death by intracellular Aβ. Finally, our results offer proof-of-concept that the clearance of intracellular Aβ by a cytosolic protease could be a viable strategy for the treatment of AD. To further evaluate the therapeutic potential of NIa, we are currently performing a series of behavioral tests on the APP_sw_/PS1 mice infused with Lenti-NIa.

We observed no apparent cytotoxicity of NIa itself *in vitro*, but did not test this issue *in vivo*. Cleavage of essential cytosolic proteins by NIa could elicit detrimental results in neuronal cells. It is intriguing to note that NIa proteases from tobacco etch virus (TEV) and tomato vein mottling virus (TVMV), the close relatives of TuMV, are frequently used for removing fusion tags from newly synthesized recombinant proteins *in vitro*. It is assumed that these proteases seldom cleave mammalian proteins due to their high substrate specificities. Nonetheless, vigorous biochemical and behavioral tests are warranted to address whether NIa is cytotoxic by itself.

## Materials and Methods

### Antibodies and reagents

Cell culture reagents were purchased from GIBCO-BRL (Invitrogen, Carlsbad, CA, USA). Synthetic Aβ_1−42_ peptide was purchased from Sigma (St Louis, MO, USA) and Anygen (Gwangju, Korea). 6E10 antibody recognizing residues 1−17 of Aβ peptide was purchased from Signet™ (Dedham, MA, USA). Antibodies against HA, α-tubulin, VDAC2, Oct1, and cathepsin D were purchased from Abcam (Cambridge, MA, UK). Chitin beads were purchased from New England BioLabs (Ipswich, MA, USA). All other reagents were purchased from Sigma.

### Purification of the NIa protease

To produce recombinant NIa protein in *E. coli*, the NIa gene was cloned into pTYB12 (New England BioLabs) via the *EcoR*I and *Xho*I sites. The pTYB12 vector contains an N-terminal intein tag. The pTYB12-NIa vector was transformed into the *E. coli* strain BL21 (DE3) and grown at 37°C in LB medium. Induction of the NIa protein was achieved by addition of 400 µM IPTG overnight at 20°C. The cells were harvested, resuspended in column buffer (20 mM HEPES [pH 7.9], 500 mM NaCl, 1 mM EDTA), and lysed by sonication. The lysate was centrifuged and the resulting supernatant was loaded onto a chitin column equilibrated with column buffer. After extensive washing, the NIa protein was eluted from the column using a column buffer containing 50 mM DTT, dialyzed in storage buffer (50 mM HEPES [pH 7.6], 1 mM EDTA, 1 mM DTT, 10% glycerol), and concentrated by Amicon Centriprep (Millipore, Billerica, MA, USA). The protein concentration was determined by the BCA method and analyzed on a 12% SDS-PAGE gel.

### Aβ preparation

To prepare Aβ solutions, we followed the method described by Yan *et al.* [41] and Dahlgren *et al.* [3]. Synthetic human Aβ_1−42_ peptides (>95% pure by high performance liquid chromatography and mass spectrometry tests) were dissolved in dimethylsulfoxide (DMSO) to a concentration of 5 mM. For monomeric Aβ, the Aβ solution in DMSO was diluted in PBS to a final concentration of 25 µM immediately before use. For oligomeric Aβ, the Aβ solution in DMSO was diluted in PBS to a concentration of 100 µM and incubated at 4°C for 36 hrs. The physical state of Aβ was confirmed by PAGE with 10−20% Tris-Tricine gels (Bio-Rad, Hercules, CA, USA).

### Cleavage assays and mass spectrometry

1.5 µM of the recombinant NIa protease was incubated with 2.5 µM Aβ preparations in an assay buffer (HEPES [pH 7.4], 20 mM KCl, 20 mM MgCl_2_) at 25°C for 3 hrs. As a control, the NIa protease was pre-incubated with the cysteine protease inhibitor, N-ethylmaleimide (NEM) for 10 min at 4°C. After incubation, the mixtures were subjected to PAGE with 10−20% Tris-Tricine gel and Western blotting using the anti-Aβ antibody 6E10. To further analyze the cleavage products, the reaction mixtures were analyzed by MALDI-TOF/TOF mass spectrometry (4700 Proteomics Analyzer, Applied Biosystems, Carlsbad, California, USA). As controls, NIa and Aβ were separately analyzed.

### Cell culture, transfection and Aβ treatment

B103 rat neuroblastoma cells were cultured in DMEM supplemented with 10% (vol/vol) fetal bovine serum[42]. A mutant NIa gene in which Asp^81^ in the catalytic triad was changed to Ala was generated by a PCR mutagenesis. To express the wild type and mutant NIa in B103 cells, the corresponding genes were subcloned into pcDNA3 (Invitrogen) containing an N-terminal HA tag. Cells were transfected using Lipofectamine Reagent (Invitrogen) according to the manufacturer's protocol. A cytosolic Aβ_1−42_ expression vector (pGFPUb-Aβ_1−42_) was previously described[25]. For the Aβ treatment, the Aβ solutions (100 µM) were added to a final concentration of 5 µM.

### Assessment of cell death

Cell viability was assessed by MTT assay and cell morphological methods. The 3-[4,5-dimethylthizaol-2-yl]-2,5-diphenyl tetrazolium bromide (MTT) was solubilized in PBS to 5 mg/ml. A volume of MTT solution equal to 10% of the culture media volume was added to the cell culture at 37°C for 3 hrs. A solubilization solution (10% Triton X-100 and 0.1 N HCl in anhydrous isopropanol) in a volume equal to the culture media volume was added and further incubated at 37°C until the resulting formazan crystals were completely dissolved. The absorbance of the samples was measured at 570 nm, and the background absorbance of each well was measured at 690 nm. For the assessment of cell morphology, cultured cells were co-transformed with the experimental plasmid and a GFP plasmid and the morphology of GFP-positive cells was examined under a fluorescence microscope[42](Olympus, Shinjuku, Tokyo, Japan).

### Immunofluorescence and confocal microscopy

B103 rat neuroblastoma cells were washed with PBS containing 1 mM CaCl_2_ and 1 mM MgCl_2_ and fixed for 10 min with 3.5% paraformaldehyde. The cells were permeabilized by incubation with 0.2% Triton X-100 in PBS for 10 min, blocked with 5% BSA in PBS for 1 hr, and incubated with anti-6E10 monoclonal antibody or HA monoclonal antibody for 1 hr. The fixed cells were then rinsed in PBS and incubated with Alexa 488 fluor-conjugated secondary antibody (Invitrogen) and TRITC-conjugated secondary antibody (Jackson Immunoresearch, West Grove, PA, USA) for 1 hr. For immunofluorescence microscopy, immunoreactivity was captured with a fluorescence microscope (Olympus) with a ProgRes C10^plus^camera (JENOPTIK, Goeschwitzer Strasse, Jena, Germany). Color coding was performed using the IMT i-solution software (IMT i-solution Inc., Vancouver, BC, Canada). To determine the levels of Aβ aggregation among GFP positive cells, the number of Aβ positive cells vs. GFP positive cells was counted in 20 random fields per culture. For confocal microscopy analysis, fluorescence signals were visualized using a confocal microscope (TCS SP2, LEICA, Ernst-Leitz-Strasse, Wetzlar, Germany).

### Subcellular fractionation

To determine the intracellular localization of the NIa protein, NIa- expressing cells were fractionated using protocol previously described[43]. Briefly, the cells were harvested by scraping into homogenation buffer (200 mM sucrose, 20 mM Tris [pH 7.4], 1 mM EGTA, 1 mM EDTA, 1 X complete protease inhibitor cocktail), lysed by multiple passages through a syringe with a 26-gauge needle, and ultracentrifuged at 70,000×g for 30 min at 4°C. The pellet (crude membrane fraction) was resuspended in homogenation buffer containing 0.5% Triton X-100 and sonicated for 1 min. Aliquots (50 µg) from each fraction were analyzed by Western blotting.

### Electrophoresis and Western blotting

The cells were harvested after washing three times with PBS, resuspended in RIPA buffer containing 1X protease inhibitor cocktail and sonicated briefly. The soluble protein fraction was recovered after centrifugation at 10,000×g for 30 min and separated by SDS–PAGE. Protein concentration was determined by the BCA method. For the analysis of Aβ peptides, samples were separated by electrophoresis using 10−20% Tris-Tricine gels. Proteins were then transferred onto PVDF membrane in 50 mM Tris, 192 mM glycine, and 20% methanol. Membranes were blocked with 5% non-fat milk and incubated with antibodies against 6E10, HA, α-tubulin, VDAC2, Oct1, and cathepsin D. Bands were visualized using the ECL reagent (GE Healthcare/Amersham Bioscience, Piscataway, NJ, USA) and the intensity of each band was quantified by densitometry (Bio-Rad).

### Production of lentiviruses

The cDNA fragments encoding NIa and GFP were subcloned into the pLEX-MCS lentiviral vector (Openbiosystems, Huntsville, AL,USA). The resulting recombinant plasmids were co-transformed with packing plasmids into 293T cells and the supernatants were collected. Lentiviruses were collected and concentrated by ultra-centrifugation as previously described [19], [44]. The titers of the NIa and GFP lentiviruses were estimated by measuring the amount of HIV p24 antigen using PCR.

### AD murine model and surgical procedure

Transgenic AD model mice, Tg-APPswe/PS1dE9, overexpressing human mutated APP and PS1 (APPswe/PS1dE9), were maintained in C57BL6 x C3H F1 hybrid mice, as described previously[45]. The mice were housed in normal plastic cages with free access to food and water in a temperature- and humidity-controlled environment under a 12 h light/dark cycle (lights on at 7 a.m.), and they were fed a diet of lab chow and water *ad libitum*. Tg-APPswe/PS1dE9 mice at 6.5 months of age were randomized into the Lenti-NIa (n = 9) and Lenti-GFP (n = 10) groups. The mice underwent bilateral intracerebroventricular (i.c.v.) infusion with 3 µl of Lenti-NIa lentivirus (1×10^8^ TU) or Lenti-GFP lentivirus with the same titer. After one month, the injected mice were sacrificed and perfused with 0.9% saline. The right and left hemispheres of the brain were used for histological and biochemical analyses, respectively. All experiments and animal procedures were approved by the Animal Care and Use Committee of the Ewha Womans University School of Medicine.

### RT-PCR

Total RNA was isolated with TRI reagent (Sigma) from frontal cerebral cortex tissue. Reverse-transcription was performed using ImProm II reverse-transcriptase (Promega, Madison, Wisconsin, USA) with oligo-dT priming. To detect NIa expression, PCR was performed using the NIa specific primer set: 5′-ACG AAA GAC GGC CAA TGC GGA-3′ and 5′-ACC CGA CGG TTG CGA TGC TT-3′. And for control experiment, PCR was performed using the GAPDH specific primer set: 5′- TCC GTG TTC CTA CCC CCA ATG-3′ and 5′- GGG AGT TGC TGT TGA AGT CGC-3′.

### Immunohistochemistry

The right hemisphere was post-fixed with 4% paraformaldehyde in 0.1 M phosphate buffer (pH 7.4) at 4°C overnight and were coronally cut into 40 µm-thick sections with a vibratome (Leica VT 1000S; Leica, Germany). Free-floating sections were blocked by 5% normal goat serum, 2% BSA, and 2% FBS. A biotinylated HRP system was used for color development. Anti-Aβ antibody Bam-10 (A5213) was purchased from Sigma (USA). Microscopic studies were carried out using an Oympus BX 51 microscope equipped with a DP71 camera and DP-B software (Olympus, Japan). For the quantification of plaque levels, the numbers of plaques in each region were measured using the TOMORO ScopeEye 3.6 program (Techsan Community, Seoul, Korea).

### Assessment of Aβ levels

ELISA assays for Aβ (1−42) and Aβ (1−40) levels were described in a previous study[46]. Briefly, the frontal cerebral cortex was homogenized in Tris-buffered saline (20 mM Tris and 137 mM NaCl, [pH 7.6]) in the presence of protease inhibitor mixtures (Complete Mini; Roche, USA). Homogenates were centrifuged at 100,000×g for 1 hr at 4°C, and the supernatant was used to measure the levels of Tris buffer-soluble forms of Aβ. The pellet was sonicated in 70% formic acid and centrifuged as above; the resulting supernatant was considered the formic acid extractable Aβ and collected for further analysis. The formic acid extract was neutralized with 1 M Tris-Cl buffer (pH 11) in a dilution ratio of 1∶20 before its use as previously described. The final assays were performed using Human Aβ (1−40) or Aβ (1−42) colorimetric sandwich ELISA kits (BioSource, Invitrogen) by following the manufacturer's instructions.

### Statistical analysis

Two sample-comparisons were carried out using the unpaired Student's *t* test with unequal variance, while multiple comparisons were made by one-way ANOVA followed by the Newman-Keuls multiple range test. A p value of less than 0.05 was accepted as being statistically significant. Data are presented as mean±SD.
